# New insights into the impact of microbiome on horizontal and vertical transmission of a tick-borne pathogen

**DOI:** 10.1186/s40168-023-01485-2

**Published:** 2023-03-14

**Authors:** Li-Feng Du, Ming-Zhu Zhang, Ting-Ting Yuan, Xue-Bing Ni, Wei Wei, Xiao-Ming Cui, Ning Wang, Tao Xiong, Jie Zhang, Yu-Sheng Pan, Dai-Yun Zhu, Liang-Jing Li, Luo-Yuan Xia, Tian-Hong Wang, Ran Wei, Hong-Bo Liu, Yi Sun, Lin Zhao, Tommy Tsan-Yuk Lam, Wu-Chun Cao, Na Jia

**Affiliations:** 1 Institute of EcoHealth, School of Public Health, Shandong University, 44 Wenhuaxi Street, Jinan, 250012 Shandong People’s Republic of China; 2 State Key Laboratory of Pathogen and Biosecurity, Beijing Institute of Microbiology and Epidemiology, Beijing, 100071 People’s Republic of China; 3 School of Medicine, Nankai University, Tianjin, 300071 People’s Republic of China; 4 State Key Laboratory of Emerging Infectious Diseases, School of Public Health, The University of Hong Kong, Hong Kong SAR, People’s Republic of China; 5 Laboratory of Data Discovery for Health Limited, 19W Hong Kong Science & Technology Parks, Hong Kong SAR, People’s Republic of China; 6 The Affiliated Hospital of Shandong University of Traditional Chinese Medicine, Jinan, 250014 People’s Republic of China; 7 Chinese PLA Center for Disease Control and Prevention, Beijing, 100071 People’s Republic of China; 8 Guangdong-Hongkong Joint Laboratory of Emerging Infectious Diseases, Joint Institute of Virology (Shantou University/The University of Hong Kong), Shantou, Guangdong 515063 People’s Republic of China; 9 EKIH (Gewuzhikang) Pathogen Research Institute, Futian District, Shenzhen City, Guangdong 518045 People’s Republic of China; 10 Centre for Immunology & Infection Limited, 17W Hong Kong Science & Technology Parks, Hong Kong SAR, People’s Republic of China

**Keywords:** Skin microbiome, Tick microbiome, Spotted fever group rickettsiae, Horizontal transmission, Vertical transmission

## Abstract

**Background:**

The impact of host skin microbiome on horizontal transmission of tick-borne pathogens , and of pathogen associated transstadial and transovarial changes in tick microbiome are largely unknown, but are important to control increasingly emerging tick-borne diseases worldwide.

**Methods:**

Focusing on a rickettsiosis pathogen, *Rickettsia raoultii*, we used *R. raoultii*-positive and *R. raoultii*-negative *Dermacentor* spp. tick colonies to study the involvement of skin microbiota in cutaneous infection with rickettsiae in laboratory mice, and the function of the tick microbiome on maintenance of rickettsiae through all tick developmental stages (eggs, larvae, nymphs, adults) over two generations.

**Results:**

We observed changes in the skin bacteria community, such as *Chlamydia*, not only associated with rickettsial colonization but also with tick feeding on skin. The diversity of skin microbiome differed between paired tick-bitten and un-bitten sites. For vertical transmission, significant differences in the tick microbiota between pathogenic *rickettsia*-positive and -negative tick chorts was observed across all developmental stages at least over two generations, which appeared to be a common pattern not only for *R. raoultii* but also for another pathogenic species, *Candidatus* Rickettsia tarasevichiae. More importantly, bacterial differences were complemented by functional shifts primed for genetic information processing during blood feeding. Specifically, the differences in tick microbiome gene repertoire between pathogenic *Rickettsia*-positive and -negative progenies were enriched in pathways associated with metabolism and hormone signals during vertical transmission.

**Conclusions:**

We demonstrate that host skin microbiome might be a new factor determining the transmission of rickettsial pathogens through ticks. While pathogenic rickettsiae infect vertebrate hosts during blood-feeding by the tick, they may also manipulate the maturation of the tick through changing the functional potential of its microbiota over the tick’s life stages. The findings here might spur the development of new-generation control methods for ticks and tick-borne pathogens.

Video Abstract

**Supplementary Information:**

The online version contains supplementary material available at 10.1186/s40168-023-01485-2.

## Introduction

Ticks (suborder: Ixodida) are blood-feeding vectors transmitting the greatest variety of pathogens [1]. Emerging tick-borne diseases account for about 75% of vector-borne diseases (https://www.who.int/news-room/fact-sheets/detail/vector-borne-diseases). For example, spotted fever group rickettsiae (SFGR) causing mild to fatal illness are nationally notifiable diseases in the USA where the annual incidence increased from 1.7 cases per million in 2000 to 13.2 in 2016 [2]. The interaction between microbiome and pathogen in ticks is just beginning to be appreciated [3–6], which is promising for new attempt to biocontrol of pathogens in vectors, as exemplified in mosquitoes [7]. However, the impact of microbiome on pathogen cutaneous infection through blood-feeding of ticks on animals, that is horizontal transmission of pathogen, and on pathogen transovarial and transstadial maintenance in ticks, that is vertical transmission of pathogen, are largely unknown. This knowledge gap is mainly caused by restricted access to the time-consuming maintenance of tick colonies of pathogen-infected vs uninfected progenies across all developmental stages.

Blood-feeding at the cutaneous interface plays a key role in vector-borne pathogen transmission. Compared with mosquitos within minutes blood meal, ticks have continuous attachment to the host skin for days [8, 9]. The skin is also the site of multiplication and persistence of bacteria such as pathogenic rickettsiae [10] and Lyme borreliosis agents [11]. The skin acts as a first line of physical and immunological defense and the skin immune system relies profoundly on its resident microbiota [12]. Although the skin microbiome is an interesting cutting-edge area [13], the interaction of vectors and skin microbiome is largely unknown.

Rickettsiae are particularly interesting in ticks since some evolved as true pathogens while others are endosymbionts [14]. The factors impacting transmission of pathogenic rickettsiae during the period from tick attachment to the host through blood feeding are unclear. We assume that host skin microbiome may regulate pathogenic rickettsia cutaneous amplification. In addition, tick-borne rickettsiae are maintained in nature mainly through vertical transmission, consisting of transovarial and transstadial passage through the female to its offspring via the egg or from one stage to another after molting, and their vertical transmission parameters are different among species [15–17]. However, the knowledge on the role of microbial population to pathogenic rickettsiae vertical transmission is also largely missing.

Here, we focused on a pathogenic SFGR species, *Rickettsia raoultii*, which causes a variable range of clinal spectrum from subclinical infection to severe complications [18]. It is mainly transmitted by *Dermacentor* spp. ticks in Europe and Asia [19]. We established *R. raoultii* -positive and -negative *Dermacentor* spp. colonies over two generations, to evaluate the role and interaction of microbial communities in host skin and ticks in vertical and horizontal transmission of pathogenic rickettsiae.

## Results

**Fig. 1:**
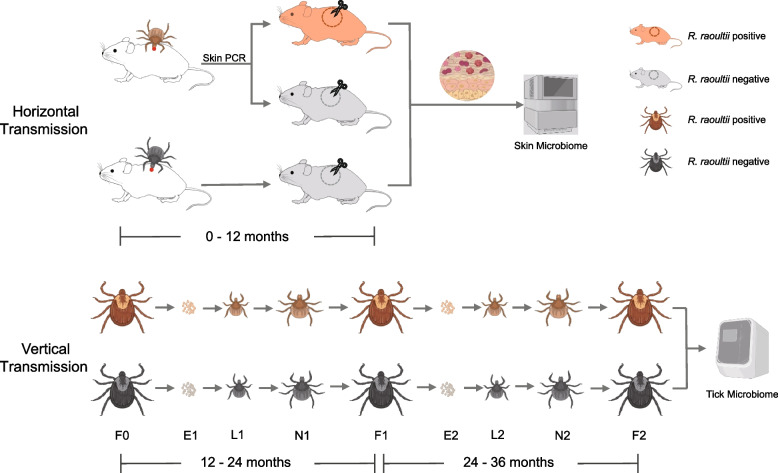
The experimental design for microbiome analysis of horizonal and vertical transmission of *Rickettsia raoultii*. In the horizontal transmission, two *R. raoultii-*positive cohorts and one negative cohort of *Dermacentor marginatus* nymphs fed on SPF BALb/c mice, including 8 mice for the first positive colony (colony 1), 8 for another positive colony (colony 2) and 8 for the negative colony (colony 3). Each Balb/C mice was fed by around 15–20 nymphs from the same cohort. In the vertical transmission, *R. raoultii* positive (*n* = 6) and negative (*n* = 6) *D. silvarum* cohorts were used. The 12-month timescales indicate the experiments carried out during the 3 years

To understand the impact of skin microbiota on localization of pathogenic *rickettsia* species from tick vector to skin of host through blood-feeding, we used *R. raoultii*-positive and *R. raoultii*-negative *Dermacentor marginatus* nymphs to feed on Balb/C mice. The skin biopsies of tick-bitten sites were evaluated for *Rickettsia* infection and microbial community (Fig. 1). We also established the vertical transmission model of pathogenic *Rickettsia* species to compare with above horizontal transmission (Fig. 1). Because of low egg hatch rate of *D. marginatus* engorged ticks, we used another *Dermacentor sp.* tick in the vertical transmission experiment, that is *D. silvarum*. Both the first (F1) and second generations (F2) of *R. raoultii*-positive and *R. raoultii*-negative *D. silvarum* tick cohorts from eggs to adults were used. The impact of tick microbiome on *R. raoultii* transmission through the full life cycle of tick was analyzed (Fig. 1). We also extended our evaluation to another pathogenic *Rickettsia* species using different tick cohorts to observe some common pattern of vertical transmission.

### Limited horizontal transmission of *R. raoultii* on the skin of Balb/C mice

**Fig. 2:**
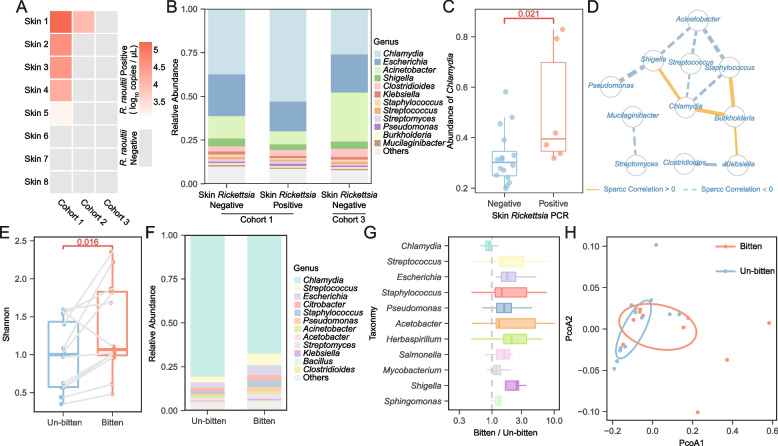
The impact of skin microbiome on pathogenic SFG rickettsial horizontal transmission. **A** *R. raoultii* load in the skin of Balb/C mice fed by positive (cohorts 1 and 2) or negative tick cohorts (cohort 3) respectively. The grey represents *R. raoultii* negative in skin while the gradient red represents positives with different bacterial loads. **B** The relative abundance of skin bacteria at genus level from different groups of Balb/C mice fed by *R. raoultii* positive or negative tick cohorts. *R. raoultii* positive vs negative skins from Balb/C mice fed by the same positive cohort 1 were also compared. **C** The relative abundance of *Chlamydia* increased significantly in skins with *R. raoultii* positive transmission than in ones with negative. **D** Correlation network of 12 most abundant bacterial genera. The thickness of lines represents the values of R square. **E** Comparison of alpha diversity (the Shannon index) of skin microbiome between paired tick-bitten and unbitten sites. **F** The relative abundance of skin microbiome between paired tick-bitten and unbitten sites. **G** The significantly altered skin bacteria between paired tick-bitten and unbitten sites. **H** PCoA of skin microbiome between paired tick-bitten and unbitten sites

Two cohorts of infected *D. marginatus* nymphs were the progenies of two engorged positive females infected with *R. raoultii*, while the un-infections were the progenies of one negative female reared under laboratory conditions. *R. raoultii*-positive colonies was confirmed by SFGR specific PCR and sequencing (Supplemental Figure S1). Each Balb/C mice was fed by around 15–20 nymphs from the same colony, including 8 mice for the positive colony No. 1 ticks, 8 for the positive colony No. 2 and 8 for the negative colony. *D. marginatus* nymphs would like to attach on ears of mice. We detected *R. raoutii* infection by qPCR and found that only 6 (37.5%) rickettsia-positive on the feeding ear of mice, mostly from the mice bitten by tick colony No.1 (Fig. 2A).

### Impact of skin microbiome on *R. raoultii* horizontal transmission

We extracted RNA from the skin biopsies of laboratory mice bitten by ticks for total RNA sequencing (meta-transcriptomics) to see the impact of skin microbiome on *Rickettsia* transmission. The cutaneous *R. raoultii*-positive (*n* = 6) and negative (*n* = 16) mice were included, among which five positives and five negatives were from the mice bitten by the same colony No. 1 ticks. A total of 12,661,715 paired reads after trimming by trim_galore and removing host reads by mapping with Balb/C mice genome were sent to Kraken 2 to obtain bacterial taxonomic classification (Supplemental Table S1).

The most abundant bacterial phyla recovered from skin biopsies (ears) of Balb/C mice were Proteobacteria (50.1%), Chlamydiae (30.5%), and Firmicutes (8.5%). The complexities of the microbiomes were unchanged by *R. raoultii* localization, however, *R. raoultii* cutaneous infection significantly altered the microbiome with an increase in the proportion of *Chlamydia* (Mann-Whitney *U* test, *p* = 0.021) (Fig. 2B, C). The significant increase of *Chlamydia* abundance explained the decrease of other genera, such as *Escherichia*, *Acinetobacter*. We also observed that *Chlamydia* abundance was higher (46.9%) in *R. raoultii-*infected skin in comparison to uninfected skin (33.9%) infested by the progeny from the same *D. marginatus* colony (colony No. 1) (Fig. 2B). In addition, a network analysis, based on co-occurrence was performed to uncover potential interactions between members of the microbial community. Of note, *Chlamydia* was the node with the most connection in the network with a total of four connections (Fig. 2D).

Tick bites impact the host in the course of a long-lasting blood meal during which tick secrets various bioactive molecules in its saliva regulating pathogen transmission [20]. We would like to explore the impact of tick-biting on host skin microbiota. *D. marginatus* nymphs usually feed on both ears of mice, and it is not justify to use skin biopsies from other part of murine body as un-bitten control because microbial flora of the skin is diverse by location [21]. We established another tick colonies, *Haemaphysalis montgomeryi*. The colonies of *Hae. montgomeryi* nymphs always attach on the back of Balb/C mice. We thus used *Hae. montgomeryi* bitten (n=12) and the paired un-bitten sites (*n* = 12) of the murine back, which were all cutaneous rickettsiae negative, to obtain their skin microbial community using total RNA sequencing (Supplemental Table S1).

We compared alpha diversity, as measured by the Shannon index, between the paired tick-bitten and un-bitten sites. Alpha diversity was significantly higher in tick-bitten sites than un-bitten sites (paired Mann-Whitney *U* test, *p* = 0.016) (Fig. 2E). At the taxonomic rank of phylum and genus, distinct differences were noted in the relative abundance of bacteria between tick-bitten and un-bitten skins (Fig. 2F, Supplemental Figure 2A). We observed the relative abundance of the dominant genus *Chlamydia* significantly decreased in the tick-bitten sites (67.5%) in comparison with the un-bitten sites (80.9%) (paired Mann-Whitney *U* test, *p* = 0.016). On the contrary, ten genera including *Streptococcus*, *Escherichia*, *Staphylococcus*, *Pseudomonas*, *Acetobacter, Herbaspirillum, Salmonella, Mycobacterium, Shigella*, and *Sphingomonas* significantly increased in the tick-bitten sites than in the un-bitten sites (Fig. 2G) (paired Mann-Whitney *U* test, *p* < 0.05).

Principal coordinate analysis based on Bray-Curtis (BC) dissimilarity showed that the microbial communities structure differed by bitten and unbitten sites (Fig. 2H) (Adonis test, *p* = 0.058), and the calculated BC dissimilarities were higher in tick-bitten group than the ones in un-bitten group (Mann-Whitney *U* test *P* < 0.001) (Supplemental Figure S2B).

### The increase of *R. raoultii* load during vertical transmission in ticks

We also established *R. raoultii* vertical transmission cohorts to compare with horizonal transmission. A total of 12 laboratory reared *D. silvarum* cohorts were evaluated, including *R. raoultii*-positive (*n* = 6) and *R. raoultii*-negative (*n* = 6) cohorts from eggs (F1), larvae (F1), nymph (F1), and adults (F1) (Supplemental Table S2). To confirm the results observed in the first generation of ticks (F1), we further reared the ticks to the second generation (F2). To elucidate the effect of *Rickettsia* infection on tick development, the fitness of NI, EPI, egg hatch percentage, and molting rate were calculated. However, no significant difference was found for *R. raoultii*-positive and *R. raoultii*-negative cohorts (Supplemental Table S3).

**Fig. 3:**
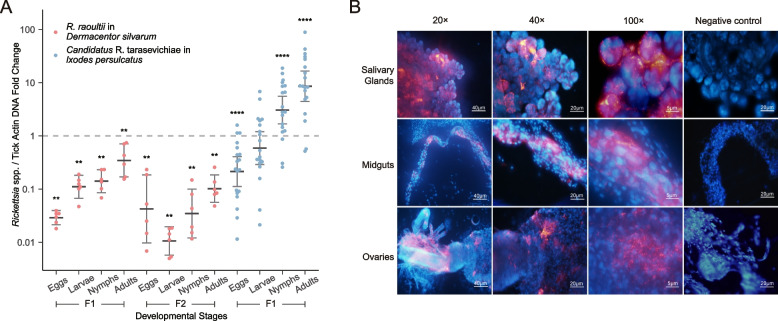
**A** The fold change of rickettsial loads in F1 or F2 generation relative to F0 female ticks during vertical transmission, which was calculated by the 2^−ΔΔCt^ method and F0 females as control (dash line) **P < 0.01, ***P < 0.001. **B** FISH detection of *R. raoultii* in different organs. Nuclei, DAPI (blue), *R. raoultii*: Quasar570 (red)

Contrast to the limited cutaneous dissemination of *R. raoultii* through horizontal transmission, we observed that the relative concentrations of *R. raoultii* clearly increased through vertical transmission from eggs to adults (Fig. 3A). (Mann-Whelopmental stages, the bacterial load of *R. raoultii* in the second generation was lower than in the first generation (Fig. 3A) (Mann-Whitney *U* test, *p* = 0itney *U* test, *p* <0.05). Furthermore, in the second generation of tick colonies (F2), although *R. raoultii* also continuously increased across the tick dev.0021 for larvae; *p* = 0.026 for nymphs; *p* = 0.009 for adults). To confirm the transovarial transmission, *R. raoultii* could be clearly seen its distribution in the ovaries and other organs (*n* = 8) (Fig. 3B).

Furthermore, we would like to depict the common pattern of pathogenic *Rickettsia* vertical transmission, thus we established another model*. Candidatus* Rickettsia tarasevichiae (CRT) is also an emerging pathogen in Asia and Europe [22]. A total of 23 laboratory reared *I. persulcatus* cohorts were evaluated, including CRT-positive (*n* = 17) and CRT-negative (*n* = 6) cohorts. We found that the egg hatch percentage was significantly higher in CRT-positive *I. persulcatus* cohorts (66.8%) than in CRT-negative cohorts (49.8%) (Mann-Whitney *U* test, *p* < 0.001) (Supplemental Table S3). The increase of CRT load was observed from eggs to adults (Fig. 3A). Previous studies reported an increasingly relative abundance of endosymbiont *Rickettsia* species through development of ticks [23], while we observed a similar pattern for the pathogenic rickettsiae.

### Impact of tick microbiome on vertical transmission of *R. raoultii* and CRT

The samples of different developmental stages from the first and second generations of laboratory-reared ticks infected or uninfected with *R. raoultii*, and from the first generation of ticks infected or uninfected with CRT, were used for bacterial 16S rRNA gene sequencing. The numbers of samples for high-throughput sequencing and the generated high-quality sequences are summarized in a supplemental table (Supplemental Table S1).

**Fig. 4:**
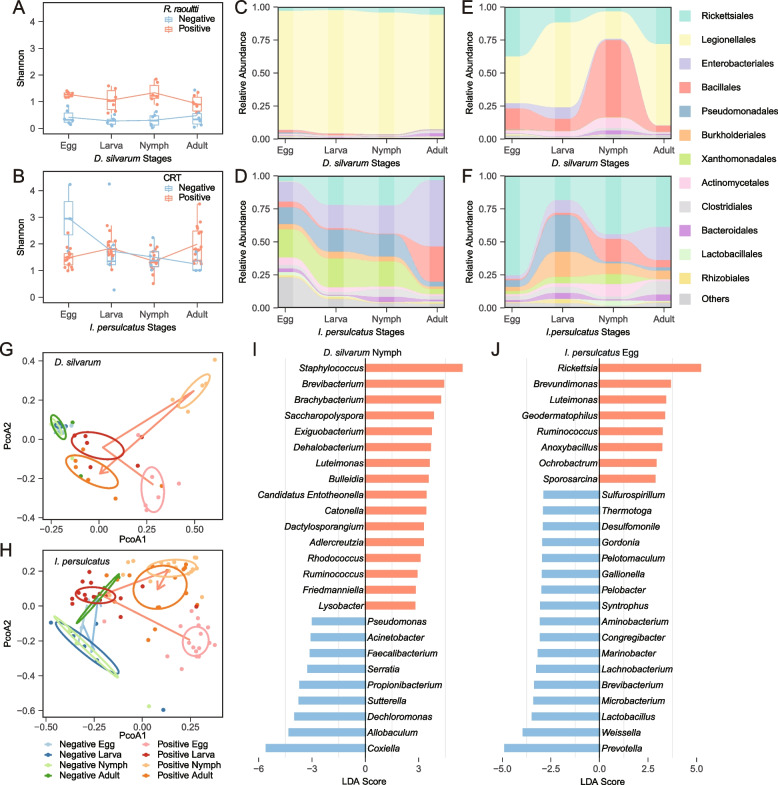
The impact of tick microbiome on pathogenic rickettsial vertical transmission (*R. raoultii* in *D. silvarum*; *Candidatus* Rickettsia tarasevichiae (CRT) in *I. persulcatus)*. The comparison of Shannon index between pathogenic SFG positive (red) and negative (blue) cohorts over all developmental stages of *D. silvarum* (**A**) and *I. persulcatus* (**B**). The relative abundance of tick microbiome across life stages in pathogenic rickettsiae-negative (**C**, **D**) and positive (**E**, **F**) cohorts. PCoA analysis of pathogenic rickettsial-positive and rickettsial-negative progenies of *D. silvarum* (**G**) and *I. persulcatus* (**H**). Histogram of LDA logarithmic scores by the linear discriminant analysis of the effect size (LEfSe analysis) comparing microbiomes in *R. raoultii*-positive and *R. raoultii*-negative nymphs of *D. silvarum* (**I**) and in CRT-positive and CRT-negative eggs of *I. persulcatus* (**J**)

The α-diversity (Shannon index) and PCoA analysis both indicated that either *R. raoultii* or CRT-positive ticks have different microbiome composition from the negative ticks (Fig. 4). For *R. raoultii*-infected tick cohorts, a significantly lower α-diversity of Shannon index were observed in *R. raoultii* negative *D. silvarum* than the positive ones (Mann-Whitney *U* test, *p* = 0.002; Fig. 4A)*.* The top 1 genus in *R. raoultii* negative cohorts was *Coxiella* (92.51%) (Fig. 4C). The second most abundant genus in those cohorts was *Rickettsia*, which should be the unclassified *Rickettsia. sp.* as another endosymbiont of *D. silvarum* [24]. The top 3 OTUs genera comprised about 89.86% of the total sequences in *R.raoultii*-positive cohorts from a full life cycle, and they were *Coxiella* (47.17%), *Staphylococcus* (21.77%), and *Rickettsia* (20.81%), which were belonged to the orders of *Legionellales, Bacillales*, and *Rickettsiales* respectively (Fig. 4E). Principal coordinate analysis based on Bray-Curtis dissimilarity indicated significant differences between the *R. raoultii*-positive and -negative groups (Adonis test, *p* = 0.001) (Fig. 4G). The second generation of *R. raoultii* cohorts had the similar results (Supplemental Figure S3).

For CRT-infected tick cohorts, although Shannon index of α-diversity was not significantly different between CRT-positive and CRT-negative groups (Mann-Whitney *U* test, *p*=0.6) (Fig. 4B), the species evenness and richness decreased from eggs to larvae, nymph and adults in CRT negative groups (Fig. 4B). The order Rickettsiales in the CRT-negative groups should be comprised of the unclassified *Rickettsia*-like endosymbiont Montezuma (Fig. 4D), which was suggested as the endosymbiont of *I. persulcatus* [25]*.* The top 3 OTUs genera *Rickettsia* (29.56%), *Pseudomonas* (8.41%) and *Stenotrophomonas* (45.05%) comprised about 43.03% of the total sequences in CRT-positive colonies, which belong to the order Rickettsiales, Pseudomonadales, and Xanthomonadakes respectively (Fig. 4F). Bray-Curtis dissimilarity found significant compositional differences between CRT-positive and CRT-negative groups (Adonis test, *p* = 0.001; Fig. 4H).

Furthermore, we would like to reveal features that most likely explained differences between *Rickettsia*-positive and negative for *D. silvarum* nymphs and *I. persulcatus* eggs with most diverse microbiome. LEfSe analysis indicated that as to *D. silvarum* nymphs*, Staphylococcus* was significantly responsible to explain differences of nymph stage in positive group (LDAscore = 5.73, *p* = 0.03), while *Coxiella* was identified to be responsible for negative cohorts at the genus taxonomic level (Fig. 4I) (LDAscore = 5.96, *p* = 0.004). For *I. persulcatus*, *Rickettsia* was significantly responsible to explain differences of eggs in positive group, while *Prevotella* was identified to be responsible for negative eggs (Fig. 4J) (LDAscore = 4.86, *p* = 0.013).

### Functional comparisons of differential microbiota in horizontal and vertical transmission

**Fig. 5:**
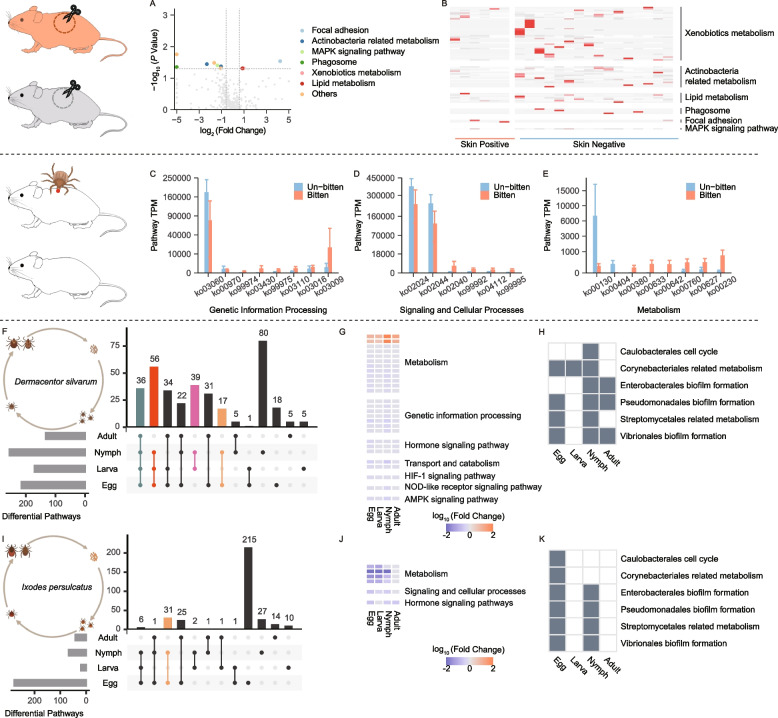
Functional shifts in the skin microbiome during pathogenic SFG rickettsial horizonal transmission (**A**–**E**) and in the tick microbiome during vertical transmission (**F**–**K**). The top KEGG pathways that were found to be differentially abundant between the skin microbiomes of *R. raoultii*-positive and *R. raoultii*-negative skins (**A**, **B**) and of paired tick-bitten and unbitten skins (**C–E**). UpSetR plot indicated the counts of differential pathways shared between pathogenic rickettsial-positive and rickettsial-negative cohorts in each developmental stage of *D. silvarum* (**F**) and *I. persulcatus* (**I**). The horizontal grey bars are the counts of differential pathways in each developmental stage. The vertical bars show the counts indicated by connected bullet points. The top KEGG pathways differentially abundant between pathogenic rickettsial positive and negative tick colonies in each stage of *D. silvarum* (**G**) and *I. persulcatus* (**J**). The differential pathways between pathogenic rickettsial positive and negative colonies of *D. silvarum* (**H**) and *I. persulcatus* (**K**) involved (grey) or not involved (white) the certain bacteria

Microbial shifts would cause alterations in the functional potential of pathogen-associated microbiome. We thus analyzed the microbiome gene repertoire for functional capabilities in pathogen-positive versus pathogen-negative cohorts in both horizontal and vertical transmission models. We observed that *R. raoultii* infection at the cutaneous interface, through blood-feeding of positive ticks, exhibited enrichment in Actinobacteria related metabolism, focal adhesion, and phagosome et al of skin microbiota (Fig. 5A, B). The abundance of Actinobacteria had strong correlation with *Chlamydia* (Mann-Kendall trend test, *p* = 0.0001) (Supplemental Figure S4). It suggested undefined interactions among *Rickettsia*, *Chlamydia*, and Actinobacteria favoring pathogenic SFG *rickettsia* horizontal transmission. Intriguingly, tick-bitten samples showed increased genetic information processing (for example protein folding, translation) and increased signaling and cellular processes (for example, signaling proteins, secretion system) of skin microbiome, suggesting counteracting reactions to salivary proteins by blood-feeding (Fig. 5C–E).

For pathogenic rickettsial vertical transmission, we found the differential KEGG pathways focused on the activity of metabolism and hormone signaling pathways for both *R. raoultii* and CRT, implying that pathogenic *rickettsia* maintenance in ticks significantly regulates microbiome function and consequently impacts on tick growth (Fig. 5F, G, I, J). Additionally, the differential KEGG pathways altered most dramatically in nymphs for *R. raoultii*-infected cohorts and in eggs for CRT-infected ticks respectively (Fig. 5F, I). In each developmental stage, certain bacterial metabolism may contribute to the functional differences between positive and negative ticks, such as Caulobacterales related metabolism for *D. silvarum* nymphs and Corynebacteriales for *I. persulcatus* eggs (Fig. 5H, K).

## Discussion

SFGR are obligate intracellular bacteria in *Rickettsia* genus of Rickettsiaceae family, and primarily transmitted to vertebrates by ticks [26]. In the past decades, human pathogenic SFGR have been increasingly identified [27, 28]. Whilst an endosymbiont rickettsia may lose its ability to enter hemocytes and salivary gland thus preventing its infecting vertebrates [29], but contributes to ticks’ fitness by supplying nutrients such as vitamins [30]. Here, we identified that pathogenic *R. raoultii* in ticks could not only actively influence skin microbiome from the feeding host to favor its infection at the cutaneous interface, but also modulate tick bacterial community to maintain its growth in ticks over a life cycle. We show that the skin microbiome, a promising research field, can indeed impact on the local dissemination of tick-borne pathogens, providing further evidence that skin is not a simple physical barrier to blood-feeding of vectors. On the other hand, compared to the uninfected cohorts, pathogenic rickettsiae infected progenies usually have a higher or more stable microbe community with functional differences mainly in metabolism and hormone signaling pathways.

The skin plays a key role in tick-borne diseases because it is the site where the tick coinoculates pathogens and its saliva, and a better understanding of what taking place in the skin should allow the development of new strategies to fight against tick-borne pathogens. Some pathogens, such as SFGR, are able to use specific skin cells and arthropod saliva for their initial development, to hide from the host immune system, and to establish persistent infection in the vertebrate host [9]. The skin microbiota has a special role on training and communication with innate and adaptive immunity of skin [21]. We observed the alteration of relative composition of skin microbiota by blood-feeding and by pathogenic SFG *rickettsia* local dissemination, illustrating close interaction between bacteria and skin immune system.

A recent work found that the domesticated amidase effector 2 (*dae2*) resisted skin-associated *staphylococci* in ticks to prevent harmful skin commensals, but had no intrinsic ability to kill tick-borne pathogen, *Borrelia burgdorferi* [31]*.* This report highlights the close contact between skin microorganisms and tick-borne pathogen. However, up to now no studies have been performed on ticks to analyze the role of host skin microbiota on tick-borne pathogen transmission [32]. In this study, we found bacteria differences with functional potential at genetic information processing such as protein folding and translation might be a counteraction of skin microbiome to a variety of tick proteins secreting in saliva during blood-feeding. Recent research also demonstrated that the skin microbiota plays an important role in the transmission of mosquito-borne pathogens [33]. Tick saliva contains an array of molecules and orchestrates a series of immune cells [9, 34]. We propose that skin bacteria may directly or indirectly modulate immune pathways and cellular processes such as focal adhesion, to creative a favorable environment for pathogenic rickettsiae surviving at the cutaneous interface. The focal adhesion mechanism has been reported to play a role in *Rickettsia * infection [34].

In the work, *R. raoultii*-positive *D. silvarum* had a more diverse microbiome than negative ones over two generations, while CRT-positive *I. persulcatus* had a more stable bacterial community than negatives across all developmental stages. It is thought that endosymbiont rickettsiae are important for tick growth and survival [35]. We found that pathogenic rickettsiae could also impact tick life. The positive ticks had diverse microbiome from negatives, with predicted gene function mainly differing at metabolism and hormone pathways. The influence of metabolites (such as cofactors and vitamins, lipid, glycan) and hormonal regulation are essential for tick development and growth [30, 36]. In general, pathogenic rickettsial species are suggested to negatively influence the fitness of their arthropod host, which is thought as a selection method that favors horizontal routes of maintenance [37]. Although overall fitness was similar in our tick colonies with or without pathogenic *Rickettsia* infection, we speculate that functional differences identified above could be used as potential markers to distinguish infected ticks from uninfected ones in future.

Interestingly, we found bacterial functional dissimilarities were lowest in *D. silvarum* adult ticks. Several research suggest that a decrease in both species richness and evenness when the tick matures from larva to adult [35, 38, 39]. It is unclear if the reduce of functional differences between *R. raoultii*-positive and *R. raoultii*-negative adult ticks perhaps reflect the requirement to maintain population stability. We should mention that the greatest functional differences were observed in egg stages of *I. persulcatus*, and egg hatch percentage was significantly different between infected and uninfected *I. persulcatus* cohorts*.* All the functional shifts identified here deserve further evaluation to dissect their exact role on tick fitness.

We suggested that the bacteria competitiveness or interaction may favor or inhibit pathogenic rickettsia transmission or maintenance. We identified the potential association of *Chlamydia* and Actinobacteria with *R. raoultii* manipulation on host skin, and of *Coxiella* with *R. raoultii* vertical transmission among ticks. In addition, Caulobacterales may specially impact on *R. raoultii* infected *D. silvarum* nymphs and Corynebacteriales on CRT-infected *I. persulcatus* eggs. *Coxiella* is the endosymbionts for *D. silvarum* [24]*.* The endosymbionts and pathogens have varied interactions, and endosymbiont can be positively or negatively correlated with pathogen [40, 41]. On the other hand, the plausible mechanism of bacteria competitiveness usually focuses on type IV, VI, and VII secretion systems [41–43], contact-dependent inhibition [44], secretion of antimicrobial products, and nutrient competition [45], which providing clues to further exploration of the bacteria interactions identified in this study.

## Conclusions

In conclusion, SFGR are important emerging tick-borne pathogens, and their horizonal and vertical transmission parameters will influence their distribution in the vector and host. Host skin microbiota should be further evaluated on its impact on tick-borne pathogen horizontal transmission. The microbiome manipulation would be a promising method for biocontrol of pathogen transmission, with more available knowledge on microbiome interaction with *rickettsia* transmission.

## Methods

### Tick collection and laboratory rearing

Tick colonies used in the horizonal transmission were *Dermacentor marginatus* and *Haemaphysalis montgomeryi* nymphs. The engorged female ticks of both species were collected from goats in Xinjiang Uygur Autonomous Region (81°54′45″E,43°34′35″N) and Yunnan Province (99°53′17″E,26°31′22″N) respectively. Tick colonies used in the vertical transmission were *Ixodes persulcatus* and *Dermacentor silvarum*. Questing *I. persulcatus* and *D. silvarum* adult ticks were collected on vegetation using blanket sweeping method in Heilongjiang Province (N44°57′43.06″，E129°13′8.32″) and Jilin Province (N43°18′36.77″E129°45′50.98″) respectively*.* SPF BALb/c mice were used for larval and nymphal feeding, while the goats were for *D. marginatus* and *H. montgomeryi* adult feeding, New Zealand white rabbits were for *D. silvarum* adult feeding and BALb/c mice were for *I. persulcatus* adult feeding respectively.

The tick colonies were reared under a 12-h light/12-h dark photoperiod at 25 °C rearing temperature in desiccators where a saturated aqueous solution of K_2_SO_4_ was used to maintain 95% relative humidity. Offspring from each female (eggs, larvae, and nymphs) were propagated in separate feeding tubes and fed on single animal.

### Pathogenic *Rickettsia*-positive and -negative tick cohort classification

We collected engorged females (F0) after completed oviposition and randomly selected subsequent hatched 3 pools of eggs (~ 100 eggs/pool) (F1egg), 3 pools of larvae (F1larva) (~ 50 larvae/pool), for pathogenic *Rickettsia* detection. Ticks were thoroughly surfaced sterilized with successive washes of 3% hydrogen peroxide, 70% ethanol twice and de-ionized H_2_O to remove environmental contamination. DNA was extracted using the Qiagen DNeasy Blood and Tissue Kit (QIAGEN, Valencia, CA, Germany) and tested by PCR for the SFGR-restricted outer membrane protein A (*omp*A) and conserved citrate synthase (*glt*A) genes as previous reports (Supplemental Table S4). All positive amplicons were sequenced to identify the *Rickettsia* species.

### Skin biopsy collection and RNA/DNA extraction

SPF BALb/c mice were fed by *D. marginatus* and *Hae. montgomeryi* nymphs respectively. Usually, 15–20 nymphs from the same cohort would feed on a single mouse. *D. marginatus* always attached on the ears while *Hae. montgomeryi* on the back. Skin biopsies of tick-bitten and un-bitten sites were collected after feeding ticks detached from the mice (around 7 days after attachment). After the mice were sacrificed, the skin biopsies were removed using sterile forceps and scissors in a clean bench to limit environmental contamination and stored at – 80 °C until use. DNA and RNA was extracted from the skin lesion using the Qiagen DNeasy Blood and Tissue Kit (QIAGEN, Valencia, CA, Germany) and RNeasy Mini Kit (QIAGEN, Valencia, CA, Germany) respectively.

### Quantification of pathogenic *Rickettsia* load in skin lesions and ticks

DNA extracted from the skin biopsies, eggs, larvae, nymphs and adults from each cohort were used to evaluate *Rickettsia* load. *Rickettsia* load was tested by real-time quantitative PCR (qPCR) targeting SFGR-specific surface cell antigen 1(sca1) gene with Roche LightCycler480II instrument (Roche, Indianapolis, IN) (Supplemental Table S4). It was calculated by the 2^-ΔΔCt^ method [46], and standard housekeeping gene (β-actin) as internal control genes. Three technical replicates were run for all experiments.

### Skin microbiome sequencing and taxonomy assignment

The extracted RNA from skin lesions was used for transcriptome sequencing (RNA-seq) after RNA quantification and quality checking. The rRNA was removed using RiBo-Zero Gold rRNA removal reagents (human/mouse/rat) (Illumina). Then the sequencing library was prepared following the Illumina standard protocol. Paired end (2 × 150 bp) sequencing of the RNA library was performed on an Illumina HiSeq 4000 platform at Novogene Tech (Beijing, China).

All host reads were mapped against Balb/C mice (GenBank assembly accession: GCA_001632525.1) using bowtie 2 [47] and removed all mapped reads by samtools [48]. The remaining reads were classified by kraken2 [49] based on NR database. The bacterial abundance was summarized at genus level, and histograms were plotted to visualize the results. Shanno index was calculated in R with the vegan package. Correlation networks were constructed by SparCC [50].

### Skin microbiome KEGG analysis

The remaining reads were assembled for each sample by SPAdes [51]. Prokka was used to accomplish gene annotation with default parameters [52]. KEGG annotation was applied by kofamscan [53]. The reads were mapped to assembly using hisat2 [54]. Transcript abundance was quantified using StringTie [55]. Transcript abundance was merged by KO of KEGG annotated above to generate the pathway abundance.

### Tick microbiome sequencing and taxonomy assignment

DNA extracted from eggs, larvae, nymphs, and engorged female adults as described above, was pooled together from each cohort according to the different development stages. Primers (515F: GTGCCAGCMGCCGCGGTAA; 806R: GGACTACHVGGGTWTCTAAT) with unique barcodes were used to amplify the V4 region of the bacterial 16S rRNA gene [4]. All ticks were amplified in triplicate and subsequently pooled to minimize PCR bias. PCR yield was assessed with a Qubit fluorometer (Life Technologies) and pooled at equimolar concentrations before a final cleanup with the QIAquick PCR purification kit (Qiagen). Amplicon libraries were prepared using Ion 520™ and Ion 530™ Kit and Single-End subsequently subject for single-end sequencing on an Ion S5 XL (Thermo Fisher Scientific, USA) following the manufacturer’s instructions. Raw FASTQ files were firstly demultiplexed and quality filtered (q20) with Quantitative Insights In Microbial Ecology (QIIME 1.9.1) [56]. All clean reads were assigned to operational taxonomic units (OTU) according to a 97% pairwise identity threshold. An open reference OTU picking strategy was used for taxonomic assignment against the GreenGenes taxonomic database (http://greengenes.lbl.gov). The relative OTU abundance tables were summarized across taxonomic level from phylum using QIIME 1.9.1.

### Downstream microbiome analysis

To characterize microbial alpha diversity, Shannon indices taking into account of both richness and evenness were calculated by using the generated genus level abundance table. Beta diversity was examined using Bray-Curtis dissimilarity. A principal coordinate analysis (PCoA) was applied to compare the distance matrices among different groups and adonis test was used to test the significance. Linear discriminant analysis of the effect size (LEfSe analysis, Segataetal., 2011) was performed at MicrobiomeAnalyst (https://www.microbiomeanalyst.ca/faces/home.xhtml) to reveal features (bacteria) that most likely explained differences between pathogenic *Rickettsia*-infected and uninfected groups. PICRUSt2 [57] was used to predict pathways based on taxonomy annotation from 16S rRNA gene sequences according to MinPath [58].

### Fitness estimates

The influence of rickettsial exposure on tick fitness was determined by calculating the nutrient index (NI) as well as the egg production index (EPI) [59]. The NI (Eq. (1)) is a measurement of efficiency of bloodmeal conversion to egg mass. The EPI (Eq. (2)) is measured to determine the efficiency with which ticks oviposit egg mass. One sample of 150 eggs was also taken from each egg clutch for the calculation of egg hatch percentage (Eq. (3)). Molting percentage was calculated for all nymph and adult ticks in selected cohorts propagated throughout the experiments. Fitness values were determined using the following equations:

Equations:

1 $$\textrm{Nutrient}\ \textrm{index}=\frac{Weight\ of\ eggs}{Initial\ weight\ of\ engorged\ tick- Residual\ weight\ of\ tick}\times 100$$

2 $$\textrm{Egg}\ \textrm{production}\ \textrm{index}=\frac{weight\ of\ eggs}{initial\ weight\ of\ engorged\ tick}\times 100$$

3 $$\%\textrm{Egg}\ \textrm{hatch}=\frac{Number\ of\ viable\ life\ stage\ }{Total\kern0.5em number\ of\ life\ stage\ prior\ to\ eclosion/ molt} \times 100$$

### Fluorescence in situ hybridization (FISH)

Tick organs were dissected in a drop of saline buffer under a stereoscopic microscope. Specimens were fixed in 3.7% (vol/vol) formaldehyde in PBS at room temperature for 10 min, then immersed in 70% (vol/vol) ethanol for at least 1 h at 4 °C to permeabilize the cells. The Probe sets (Supplemental Table S5) were designed for *R. raoultii* 16S rRNA gene labeled with Quasar®570 (Stellaris RNA FISH probes). The Stellaris RNA FISH was then carried out according to the protocol (Stellaris™ , LGC Biosearch Technologies, USA). Negative controls were established in parallel. Stained samples were whole mounted and viewed under an Olympus FluoViewDP72 confocal microscope.

## Supplementary Information

**Additional file 1: Figure S1.** Phylogenetic analysis of sequences from SFGR specific PCR with other Rickettsial specieses. Phylogenetic trees of *omp*A and *glt*A genes of SFGR identified from *I. persulcatus* cohort A) *omp*A and B) *glt*A and from *D. silvarum* C）*omp*A, D) *glt*A, respectively.

**Additional file 2: Figure S2.** Composition and similarity of bacteria on bitten and un-bitten skins. A) Relative abundance of bacteria between tick-bitten and un-bitten skins. B) Bray−Curtis dissimilarity within bitten or unbitten group and between unbitten and bitten group.

**Additional file 3: Figure S3.** PCoA analysis of *R. raoultii*-positive and -negative groups in second generation.

**Additional file 4: Figure S4.** The abundance correlation between *Chlamydia* and Actinobacteria in skin.

**Additional file 5: Supplemental Table S1.** Reads counts of data analyzed in the study.

**Additional file 6: Supplemental Table S2.** The laboratory life cycle of *Ixodes persulcatus* and *Dermacentor silvarum*.

**Additional file 7: Supplemental Table S3.** Results of tick fitness post-rickettsial exposure in *Ixodes persulcatus* and *Dermacentor silvarum*.

**Additional file 8: Supplemental Table S4.** Primers used in this study.

**Additional file 9: Supplemental Table S5.** The Probe sets were designed for *R. raoultii* 16S rRNA gene.

## Data Availability

The datasets supporting the findings of this article are available in the NCBI Sequence Read Archive (SRA) database (BioProject number: PRJNA863388, PRJNA863738, PRJNA863433).

## Abbreviations

FISH: Fluorescence in situ hybridization
SFGR: Spotted fever group rickettsiae
BC: Bray-Curtis
PCR: Polymerase chain reaction
qPCR: Real-time quantitative polymerase chain reaction
NI: Nutrient index
EPI: Egg production index
CRT: *Candidatus* Rickettsia tarasevichiae
PCoA: Principal coordinate analysis
OTUs: Operational taxonomic units
LEfSe: Linear discriminant analysis of the effect size
LDAscore: Linear discriminant analysis score
KEGG: Kyoto Encyclopedia of Genes and Genomes
*omp*A: Outer membrane protein A
*glt*A: Conserved citrate synthase
*sca*1: Surface cell antigen 1
NR: Non-Redundant Protein Sequence Database
QIIME: Quantitative Insights In Microbial Ecology
SPF: Specific pathogen free
DNA: Deoxyribonucleic acid
RNA: Ribonucleic acid
16S rRNA: 16S ribosomal RNA
K_2_SO_4_: Potassium sulfate
H_2_O: Water
DAPI: 4',6-diamidino-2-phenylindole
