# Supplementary figures and images for: New insights into the impact of microbiome on horizontal and vertical transmission of a tick-borne pathogen
Source: Microbiome. 2023 Mar 14;11:50. doi: 10.1186/s40168-023-01485-2 (PMC10012463; doi:10.1186/s40168-023-01485-2)

A

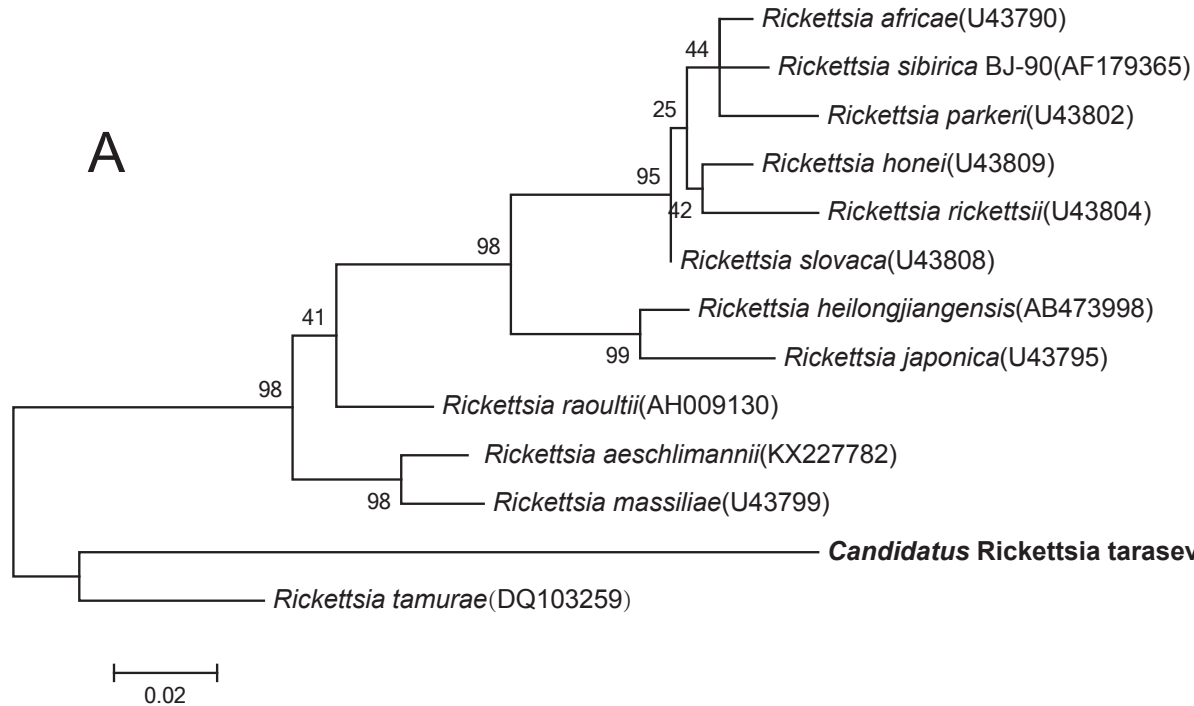

B

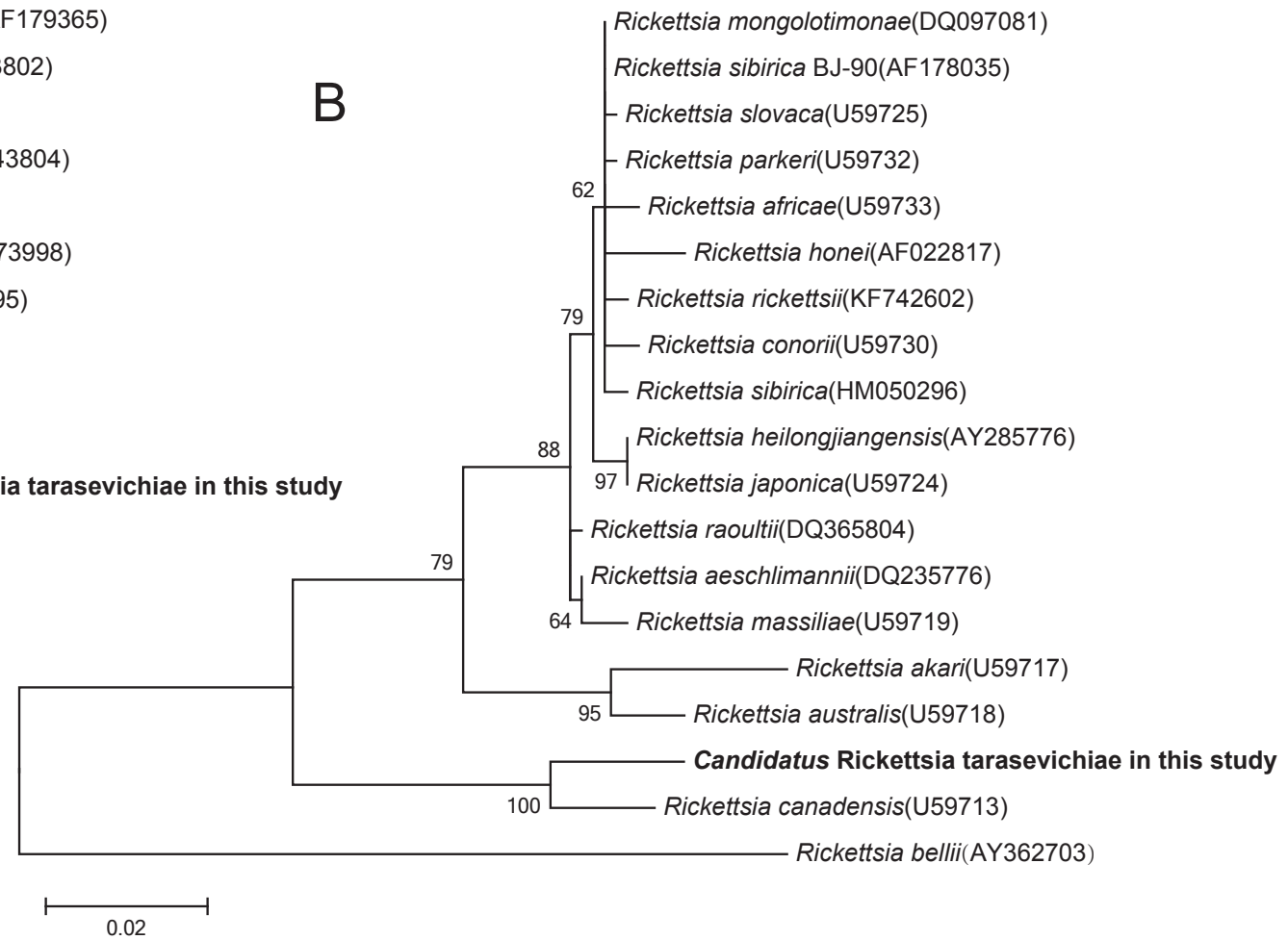

C

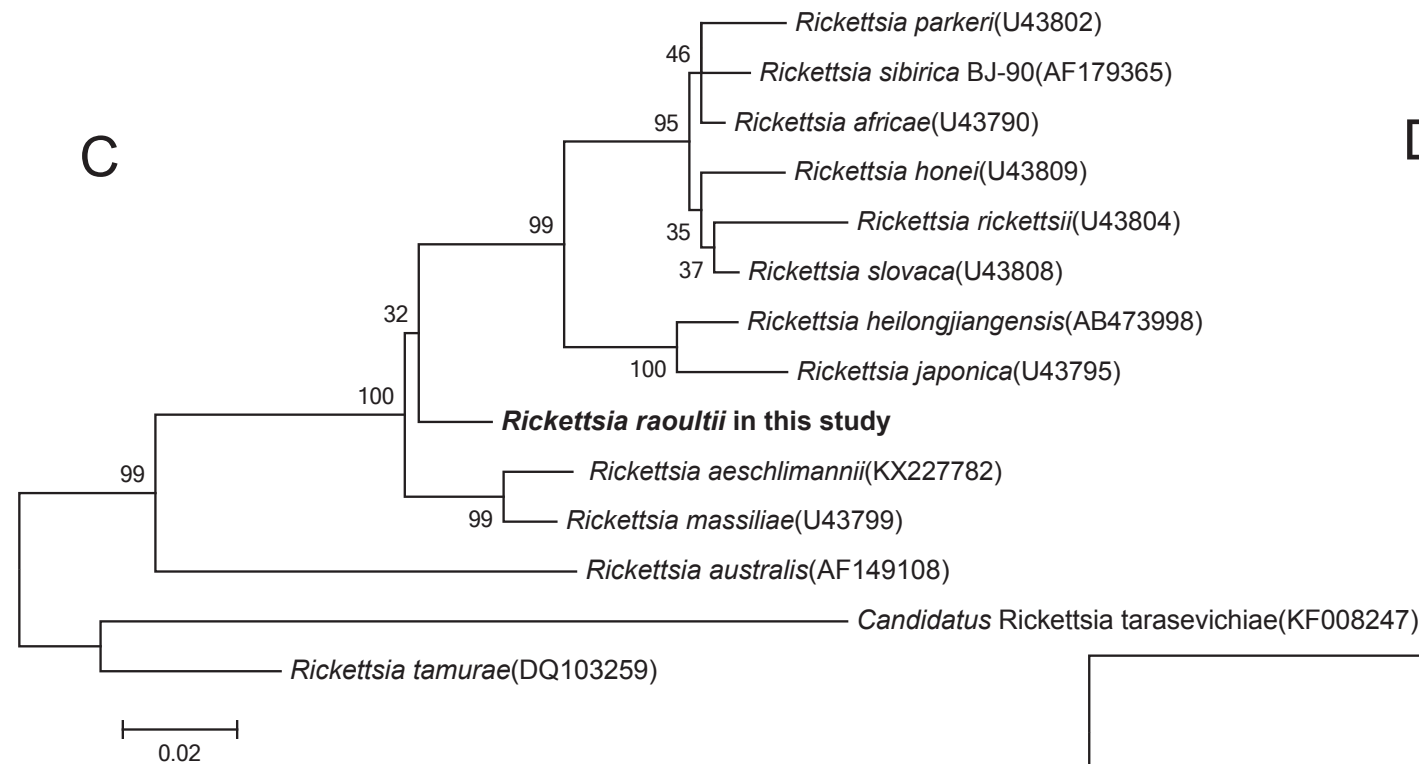

D

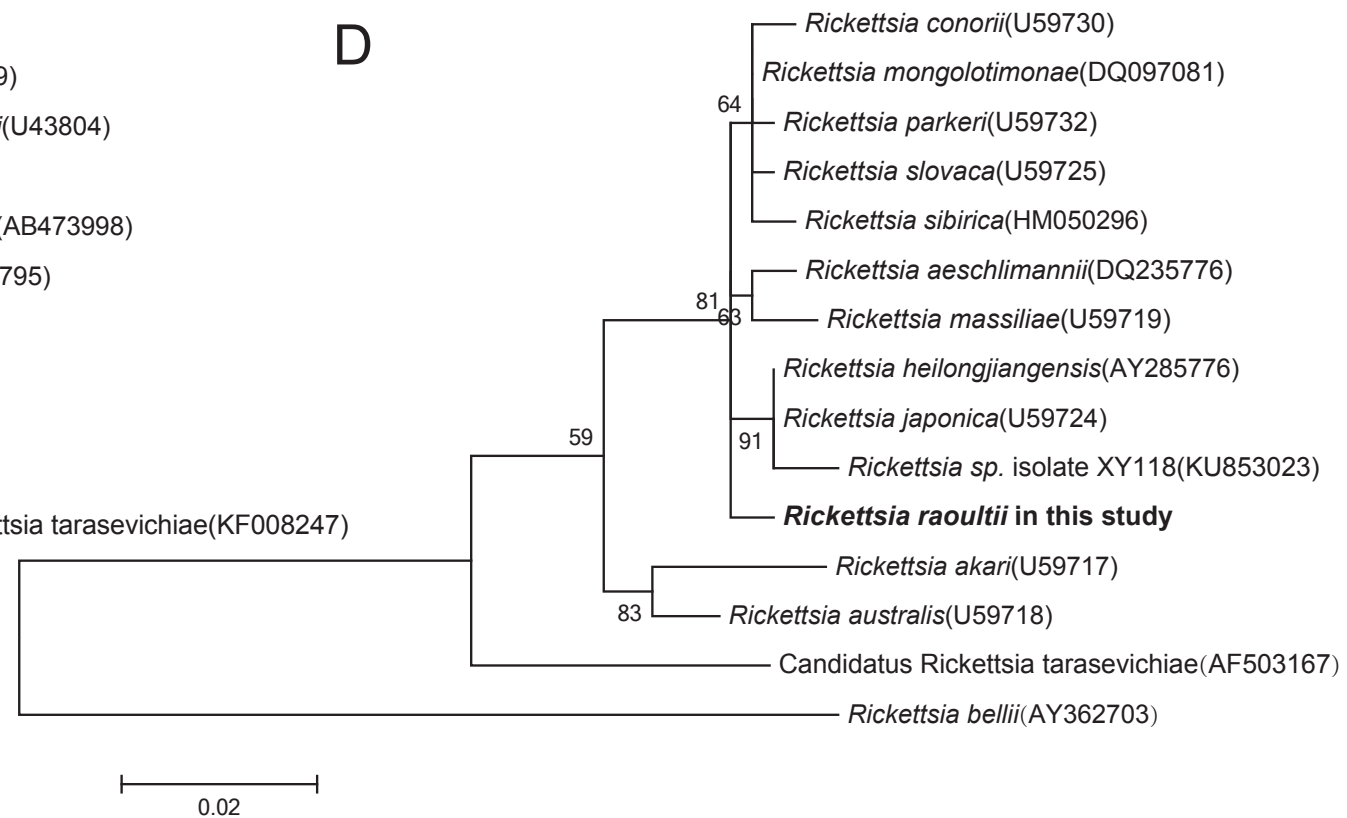

Supplement: Supplementary file 2 — Additional file 1: Figure S1. Phylogenetic analysis of sequences from SFGR specific PCR with other Rickettsial specieses. Phylogenetic trees of ompA and gltA genes of SFGR identified from I. persulcatus cohort A) ompA and B) gltA and from D. silvarum C）ompA, D) gltA, respectively. [file 40168_2023_1485_MOESM1_ESM.pdf]

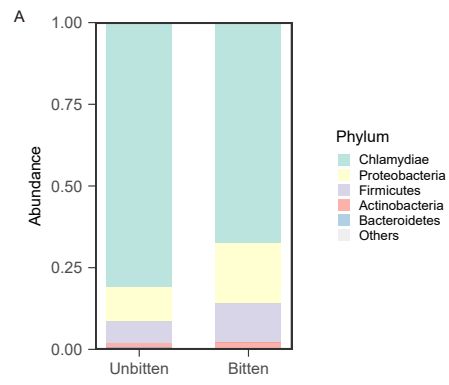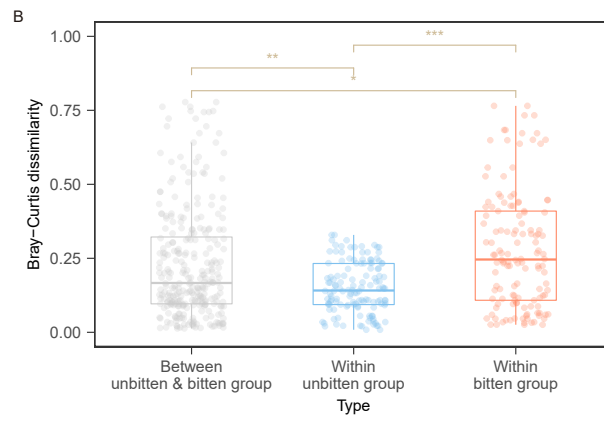

Supplement: Supplementary file 3 — Additional file 2: Figure S2. Composition and similarity of bacteria on bitten and un-bitten skins. A) Relative abundance of bacteria between tick-bitten and un-bitten skins. B) Bray−Curtis dissimilarity within bitten or unbitten group and between unbitten and bitten group. [file 40168_2023_1485_MOESM2_ESM.pdf]

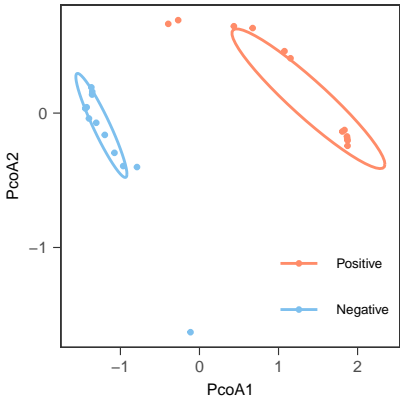

Supplement: Supplementary file 4 — Additional file 3: Figure S3. PCoA analysis of R. raoultii-positive and -negative groups in second generation. [file 40168_2023_1485_MOESM3_ESM.pdf]

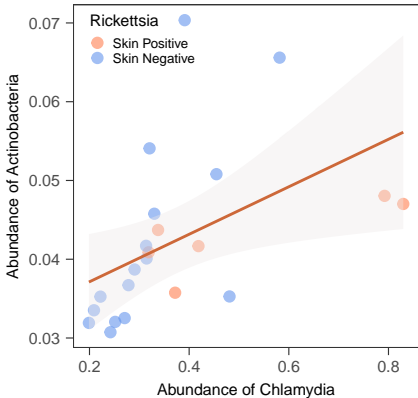

Supplement: Supplementary file 5 — Additional file 4: Figure S4. The abundance correlation between Chlamydia and Actinobacteria in skin. [file 40168_2023_1485_MOESM4_ESM.pdf]
