# Supplementary material for: New insights into the impact of microbiome on horizontal and vertical transmission of a tick-borne pathogen
Source: Microbiome. 2023 Mar 14;11:50. doi: 10.1186/s40168-023-01485-2 (PMC10012463; doi:10.1186/s40168-023-01485-2)
Supplement: Supplementary file 6 — Additional file 5: Supplemental Table S1. Reads counts of data analyzed in the study. [file 40168_2023_1485_MOESM5_ESM.docx]

| Study | Sample counts | | Cleaned reads paired counts |
| --- | --- | --- | --- |
| Horizontal transmission of *R. raoultii* on the skin of mice | 22 (6 positive vs 16 negative samples) | 12,661,715 | |
| Impact of tick-biting on host skin microbiota | 24 (12 bitten vs 12 un-bitten samples) | 10,901,457 | |
| Vertical transmission of *R. raoultii* | 48 (12 eggs, 12 larvae, 12 nymphs, 12 adults) | 2,374,394 | |
| Vertical transmission of CRT | 83 (20 eggs, 23 larvae, 21 nymphs, 19 adults) | 5,198,334 | |

**Supplemental Table 1.** Reads counts of data analyzed in the study
