# Supplementary material for: New insights into the impact of microbiome on horizontal and vertical transmission of a tick-borne pathogen
Source: Microbiome. 2023 Mar 14;11:50. doi: 10.1186/s40168-023-01485-2 (PMC10012463; doi:10.1186/s40168-023-01485-2)
Supplement: Supplementary file 7 — Additional file 6: Supplemental Table S2. The laboratory life cycle of Ixodes persulcatus and Dermacentor silvarum. [file 40168_2023_1485_MOESM6_ESM.docx]

Supplemental table 2 The laboratory life cycle of *Ixodes persulcatus* and *Dermacentor silvarum*

| Life stage | Development stage | *Ixodes persulcatus* | | | *Dermacentor silvarum* | | |
| --- | --- | --- | --- | --- | --- | --- | --- |
|  |  | Number tested | Days | Mean±SEM | Number tested | Days | Mean±SEM |
| Adults | Feeding | 49 | 5-10 | 7.28±1.66 | 12 | 7-12 | 9.08±1.61 |
|  | Preoviposition |  | 2-4 | 2.95±0.88 |  | 2-4 | 2.92±0.67 |
|  | Oviposition |  | 15-30 | 21.85±2.14 |  | 10-13 | 13.75±1.06 |
| Eggs | Prehatching | 250*  (cohort number=23) | 3-4 | 3.48±0.51 | 250*  (cohort number=12) | 2-4 | 3.17±0.72 |
|  | Hatching |  | 10-25 | 17.45±1.24 |  | 10-20 | 15.67±2.42 |
| Larvae | Feeding | 100*  (cohort number=23) | 2-3 | 2.45±0.50 | 100*  (cohort number=12) | 2-3 | 2.92±0.67 |
|  | Digesting |  | 20-45 | 32.93±2.96 |  | 3-7 | 5.08±1.31 |
|  | Molting |  | 10-15 | 12.28±1.85 |  | 10-15 | 13.75±1.48 |
| Nymphs | Feeding | 50*  (cohort number=23) | 3-7 | 4.80±1.42 | 50*  (cohort number=12) | 4-7 | 5.67±0.89 |
|  | Digesting |  | 30-65 | 48.00±2.16 |  | 10-17 | 14±1.95 |
|  | Molting |  | 35-150 | 95.00±2.67 |  | 10-23 | 15.58±2.15 |
| Life cycle |  |  | 177-278 | 208.61±8.47 |  | 76-120 | 95.67±7.29 |

* The testing number of cohorts. Cohorts created by the progeny of individual females were followed through the egg, larval, nymphal, and adult stage to assess vertical transmission.
