# Supplementary material for: New insights into the impact of microbiome on horizontal and vertical transmission of a tick-borne pathogen
Source: Microbiome. 2023 Mar 14;11:50. doi: 10.1186/s40168-023-01485-2 (PMC10012463; doi:10.1186/s40168-023-01485-2)
Supplement: Supplementary file 8 — Additional file 7: Supplemental Table S3. Results of tick fitness post-rickettsial exposure in Ixodes persulcatus and Dermacentor silvarum. [file 40168_2023_1485_MOESM7_ESM.docx]

**Supplemental Table 3 Results of tick fitness post-rickettsial exposure in *Ixodes persulcatus* and *Dermacentor silvarum***

| Development evaluation index | *Ixodes persulcatus* | | | *Dermacentor silvarum* | | |
| --- | --- | --- | --- | --- | --- | --- |
|  | Infected cohort  (n=17 cohorts)  Mean(95%CI) | Uninfected cohort  (n=6 cohorts)  Mean(95%CI) | Total  (n=23 cohorts)  Mean(95%CI) | Infected cohort  (n=6 cohorts)  Mean(95%CI) | Uninfected cohort  (n=6 cohorts)  Mean(95%CI) | Total  (n=12 cohorts)  Mean(95%CI) |
| Engorgement weight (mg) | 448.1  (405.13-491.07) | 458.7  (356.28-561.12) | 446.7  (410.73-482.67) | 565.6  (531.99-599.21) | 533.7  (512.10-555.30) | 549.7  (528.77-570.63) |
| Nutrient index (NI) (%) | 33.9(30.75-37.05) | 41.5(38.54-44.46) | 36.9(34.24-39.56) | 42.7(39.02-46.38) | 40.1(34.42-45.78) | 41.4(38.06-44.74) |
| Egg production index (EPI) (%) | 26.9(24.31-29.49) | 29.2(26.24-32.16) | 27.8(25.82-29.78) | 31.3(28.10-34.50) | 29.9(25.74-34.06) | 30.6(28.05-33.15) |
| Hatching rate(%) | 66.8(63.21-70.39)* | 49.8(45.88-53.72)* | 65.9(62.10-69.70) | 44.3(38.78-49.82) | 37.5(24.38-50.62) | 40.9(33.83-47.97) |
| Molt-nymph rate(%) | 36.3(32.71-39.89) | 36.1(29.78-42.42) | 36.2(33.58-38.82) | 60.7(52.06-69.34) | 60.1(51.46-68.74) | 60.4 (54.57-66.23) |
| Molt-adult rate(%) | 17(14.11-19.89) | 14.3 (9.02-19.58) | 15.8(13.35-18.25) | 84.0(78.16-89.84) | 80.3(69.98-90.62) | 82.2(76.43-87.97) |

* Mann-Whitney U test *P*<0.001.
