# Supplementary material for: New insights into the impact of microbiome on horizontal and vertical transmission of a tick-borne pathogen
Source: Microbiome. 2023 Mar 14;11:50. doi: 10.1186/s40168-023-01485-2 (PMC10012463; doi:10.1186/s40168-023-01485-2)
Supplement: Supplementary file 9 — Additional file 8: Supplemental Table S4. Primers used in this study. [file 40168_2023_1485_MOESM8_ESM.docx]

| Supplemental Table 4 Primers used in this study | | | | |
| --- | --- | --- | --- | --- |
| Organism | Target Gene | Primer | Nucleotide Sequence(5'-3') | Reference |
| Spotted fever group rickettsiae | *ompA* | Rr190.70f | ATGGCGAATATTTCTCCAAAA | ([Roux et al. 1996](#_ENREF_2)) |
|  |  | Rr190.701r | GTTCCGTTAATGGCAGCATCT |  |
|  |  | Rr190.602r | AGTGCAGCATTCGCTCCCCCT |  |
|  | *gltA* | CS2d | ATGACCAATGAAAATAATAAT | ([Bennett 1974](#_ENREF_1)) |
|  |  | CSEndr | CTTATACTCTCTATGTACA |  |
|  |  | RpCS877 | GGGGACCTGCTCACGGCGG |  |
|  |  | RpCS1258 | ATTGCAAAAAGTACAGTGAACA |  |
|  | sca1 | Sca1 | GTTTGTGGATGCGTGGTA |  |
|  |  | Sca134 | AACCCGATAGTAGCAC |  |
|  | β-actin | β-actin F | AAGGACCTGTACGCCAACAC | 196bp |
|  |  | β-actin R | ACATCTGCTGGAAGGTGGAC |  |

Bennett, G.F. (1974) Oviposition of boophilus microplus (canestrini) (acarida: Ixodidae). Ii. Influence of temperature, humidity and light. Acarologia 16, 251-7

Roux, V., P.E. Fournier, D. Raoult (1996) Differentiation of spotted fever group rickettsiae by sequencing and analysis of restriction fragment length polymorphism of pcr-amplified DNA of the gene encoding the protein rompa. J Clin Microbiol 34, 2058-65
