# Supplementary material for: New insights into the impact of microbiome on horizontal and vertical transmission of a tick-borne pathogen
Source: Microbiome. 2023 Mar 14;11:50. doi: 10.1186/s40168-023-01485-2 (PMC10012463; doi:10.1186/s40168-023-01485-2)
Supplement: Supplementary file 10 — Additional file 9: Supplemental Table S5. The Probe sets were designed for R. raoultii 16S rRNA gene. [file 40168_2023_1485_MOESM9_ESM.docx]

**Supplemental Table 5 The Probe sets were designed for *R. raoultii* 16S rRNA gene**

| Sequence | Sequence Name |
| --- | --- |
| TAAGCATACCGATAGCGTTC | raoultii16S_1 |
| CAATTAGTCCGTTCGACTTG | raoultii16S_2 |
| GCCACTAACTAATTGGAGCA | raoultii16S_3 |
| GATTCCCACGTGTTACTCAC | raoultii16S_4 |
| AGTTATTCCGTACTGATGGG | raoultii16S_5 |
| TCCGTAGAGAATATGCGGTA | raoultii16S_6 |
| GCTCATCCATCAGCGATAAA | raoultii16S_7 |
| TACCTCACCAACTACCTAAT | raoultii16S_8 |
| CAGATCGTCGGCTTGGTAAG | raoultii16S_9 |
| TGATCATCCTCTCAGACCAG | raoultii16S_10 |
| CATTGTCCAATATTCCCCAC | raoultii16S_11 |
| TATTGCTGGATCAGGCTTTC | raoultii16S_12 |
| TAAGGCCTTCATCACTCACT | raoultii16S_13 |
| TCTTCCTTGCTAAAAGAGCT | raoultii16S_14 |
| GGGCTTTTTCTGCAAGTAAC | raoultii16S_15 |
| CCAGTAATTCCGAACAACGC | raoultii16S_16 |
| CTTCCAACTTACTAAACCGC | raoultii16S_17 |
| AAGCAATTCCGAGGTTAAGC | raoultii16S_18 |
| CCCTACTACACTCTAGATTA | raoultii16S_19 |
| ATTTCACCTCTACACTAGGA | raoultii16S_20 |
| GTGTTCCTCCTAATATCTAA | raoultii16S_21 |
| TGCATCAGCGTCAGTTGTAG | raoultii16S_22 |
| TATCTAATCCTGTTTGCTCC | raoultii16S_23 |
| TTTACGGCGTGGACTACCAG | raoultii16S_24 |
| TTCCGATATCTAGCACTCAT | raoultii16S_25 |
| AGCTGCGAAACCGAAAGAGA | raoultii16S_26 |
| CAGGCGGAGTGCTTAATGCG | raoultii16S_27 |
| GTTTTAATCTTGCGACCGTA | raoultii16S_28 |
| ATTAAACCGCATGCTCCACC | raoultii16S_29 |
| AAGGTTTTTCGCGTAACATC | raoultii16S_30 |
| GACCACCATGTCAAGGGTTG | raoultii16S_31 |
| AGCCGAGCTGAAGAAAAGCA | raoultii16S_32 |
| CATGCAACACCTGTGTGTGG | raoultii16S_33 |
| TCACGACACGAGCTGACGAC | raoultii16S_34 |
| TTGCGGGACTTAACCCAACA | raoultii16S_35 |
| ACCCGCTGGCAAATAAGAAT | raoultii16S_36 |
| CGGCAGTTTTCTTATAGTTC | raoultii16S_37 |
| TAAGGGCCATGATGACTTGA | raoultii16S_38 |
| CATTGTAGCACGCGTGTAGC | raoultii16S_39 |
| TCTTGCTTCCCTCTGTAAAC | raoultii16S_40 |
| TGTCTTTTAGGGATTTGCTC | raoultii16S_41 |
| GCAGAGAACAATCCGAACTG | raoultii16S_42 |
| ATTCCAACTTCATGCTCTCG | raoultii16S_43 |
| TGCTGATCCGCGATTACTAG | raoultii16S_44 |
| CGAGAACGTATTCACCGCGG | raoultii16S_45 |
| TGACGGGCAGTGTGTACAAG | raoultii16S_46 |
| TTCAGGTAAAACCAACTCCC | raoultii16S_47 |
| CAGTCGCTAATTTTACCGTG | raoultii16S_48 |
| P48 Mix | raoultii16S |
